# When perception is stronger than physics: Perceptual similarities rather than laws of physics govern the perception of interacting objects

**DOI:** 10.3758/s13414-021-02383-1

**Published:** 2021-10-18

**Authors:** Alexander Pastukhov, Lisa Koßmann, Claus-Christian Carbon

**Affiliations:** 1 Department of General Psychology and Methodology, University of Bamberg, Markusplatz 3, D-96047 Bamberg, Germany; 2 Forschungsgruppe EPÆG (Ergonomics, Psychological Æsthetics, Gestalt), Bamberg, Bavaria Germany

**Keywords:** Multistable perception, Prior knowledge, Binocular rivalry, Vision, Perceptual inference

## Abstract

When several multistable displays are viewed simultaneously, their perception is synchronized, as they tend to be in the same perceptual state. Here, we investigated the possibility that perception may reflect embedded statistical knowledge of physical interaction between objects for specific combinations of displays and layouts. We used a novel display with two ambiguously rotating gears and an ambiguous walker-on-a-ball display. Both stimuli produce a physically congruent perception when an interaction is possible (i.e., gears counterrotate, and the ball rolls under the walker’s feet). Next, we gradually manipulated the stimuli to either introduce abrupt changes to the potential physical interaction between objects or keep it constant despite changes in the visual stimulus. We characterized the data using four different models that assumed (1) independence of perception of the stimulus, (2) dependence on the stimulus’s properties, (3) dependence on physical configuration alone, and (4) an interaction between stimulus properties and a physical configuration. We observed that for the ambiguous gears, the perception was correlated with the stimulus changes rather than with the possibility of physical interaction. The perception of walker-on-a-ball was independent of the stimulus but depended instead on whether participants responded about a relative motion of two objects (perception was biased towards physically congruent motion) or the absolute motion of the walker alone (perception was independent of the rotation of the ball). None of the two experiments supported the idea of embedded knowledge of physical interaction.

**Supplementary Information:**

The online version contains supplementary material available at 10.3758/s13414-021-02383-1.

Most of the time, our perception is stable and unambiguous, in the sense that a single physical stimulus leads to a singular stable perception. However, *multistable* displays, which are compatible with several comparably likely perceptual interpretations, lead to unstable perception, as their continuous viewing leads to repeated perceptual switches between the alternatives. When several identical or similar multistable displays are viewed together, their perception tends to be synchronized, and they tend to be in the same dominant perceptual state most of the time (Eby et al., 1989; Ramachandran & Anstis, 1983). This phenomenon is called *perceptual coupling* and the perceptual synchronization is thought to reflect a bias from a perceptually dominant state that could be implemented either via local mechanisms (Klink et al., 2009) or top-down feedback (Grossmann & Dobbins, 2003).

The strength of the bias primarily reflects the similarity of displays and their proximity (Adams & Haire, 1958; Eby et al., 1989; Grossmann & Dobbins, 2003). However, prior research suggested that the perception of coupled displays may reflect embedded knowledge about statistical regularities of physical interaction, at least for some specific types of displays and for their specific layouts. For example, it has been argued that the tendency of kinetic-depth effect displays to corotate may reflect a “common spin” assumption, particularly when the objects are placed coaxially (Dobbins & Grossmann, 2018). When two kinetic-depth objects are positioned so that they are touching, their perception could be biased towards counter-rotation by embedded knowledge of frictional interaction (Gilroy & Blake, 2004) but see (Pastukhov, Zaus, et al., 2018b). The same friction prior is also hypothesized to ensure a physically congruent perception of a walker on a ball, as the latter rolls under their feet (Jackson & Blake, 2010). The idea of context-specific physics-based interaction is supported by other experiments, such as a streaming-bouncing display. Here, two identical or similar objects move through each other along a linear trajectory and appear to either “stream” through each other or “bounce” off each other (Burns & Zanker, 2000). The latter perception is facilitated by a sound that is thought to serve as auditory evidence for the collision and, therefore, bias perception towards the bounce (Scholl & Nakayama, 2002).

Our knowledge about such embedded statistical regularities, irrespective of how they are represented in the neural network of the brain (Rideaux & Welchman, 2020), and the specific context they are used in, would help us better understand the process of perceptual inference and predict situations when similar priors might be at play. Therefore, we sought to replicate and extend prior work on the possible role of an embedded knowledge of physical interaction in the perception of bistable kinetic-depth displays. To this end, we employed two displays. For the first experiment, we designed a novel display with two ambiguously rotating gears that can interact via interlocking. In the second experiment, we used the walker-on-a-ball display, as used by Jackson and Blake (2010). Importantly, in both cases, a stimulus configuration that allows for a physical interaction reliably leads to the perception that is consistent with it (i.e., the gears counterrotate and the ball rolls under the walker’s feet in a physically consistent direction). For both displays, we utilized a variety of gradual stimulus manipulations that were designed to either introduce abrupt changes to the potential physical interaction between objects or keep it constant despite changes in visual stimulus (distance between objects, their ambiguity, etc.) To quantify potential influences, we fitted the participant’s perceptual data using four different models. An *independent-perception,* effectively, a null model assumed that the perception was independent of the stimulus manipulation and reflected only an intrinsic sensory bias of a participant (Wexler et al., 2015). A *stimulus-based* model presupposed that gradual display changes should lead to similarly gradual changes in perception. A *physics-based* model predicted that the perception should depend only on whether a display allowed for physical interaction. Finally, a *hybrid-interaction* model assumed that participants’ perception depended on both properties of the visual display and interaction between visual display properties and a possibility of physical interaction. In other words, the hybrid-interaction model assumed that visual display properties had a different effect on perception depending on whether the stimulus allowed for the physical interaction.

We report that the perception of the ambiguous gears was consistent with a purely *stimulus-based* model. In contrast, for the walker-on-a-ball, the perception was best described by the independent-perception model as it depended neither on stimulus properties nor on the possibility for physical interaction. Thus, neither experiment provided evidence for the embedded physics of interaction.

## Method

### Participants

Participants were recruited through advertisements posted around the University of Bamberg. Consequently, our sample consisted predominantly of young university students, making it comparable to a typical population sample in other studies on multistable perception. We limited our sample size to 20 participants based on the power analysis that was part of the preregistration (see https://osf.io/2aksm).

Twenty participants (16 females, four males; age range: 19–56 years) took part in Experiment 1, and 19 in Experiment 2 (one participant could not finish the experiment due to an attack of migraine). Ten participants (nine females, age range: 20–24 years, one male 23 years old) took part in an additional experimental condition for Experiment 2. The 11th participant was planned but could not participate in the additional experimental condition for Experiment 2 because all testing was immediately canceled due to the SARS-CoV-2 outbreak. Due to a software error, only one of the conditions was saved for four participants (DTS1997MRNO, ILM1998WRNO, JTB1998WRNO, SKM2000WLNO) in the additional experimental condition.

All procedures were in accordance with the national ethical standards on human experimentation and with the Declaration of Helsinki of 1975, as revised in 2008. The study was in full accordance with the ethical guidelines of the University of Bamberg and was approved by an umbrella evaluation for psychophysical testing of the university ethics committee (Ethikrat) on 18 August 2017. Informed consent was obtained from all observers prior to each experimental session. All participants had normal or corrected-to-normal vision and normal color vision, all tested by standard tests in situ, and were naïve to the purpose of the study. For their participation, observers received credit within the framework of a mandatory module of research participation in accordance with the standards of the University of Bamberg.

### Apparatus

Displays were presented on a 61.0 cm diagonal EIZO CG245W screen with the size of the visible area 51.7 cm × 32.3 cm, resolution 1,920 × 1,200, refresh rate 60 Hz. A continuous viewing distance of 65 cm was ensured by chin and forehead rests that stabilized the viewing position and angle. This experimental setup means that a single pixel subtended 0.023° of visual angle.

### Experiment 1. Gears

The participants viewed two rotating gears for 1 minute and indicated the direction of rotation of the gears by continuously pressing one of four corresponding arrow keys (see below). The gears were 7 dva (degrees of visual angle) in diameter and had 24 teeth each. One of the gears was rotated by 7.5° so that they appeared to interlock when overlapping. The gears were rotating at a speed of 0.71 Hz/255°/s.

**Fig. 1:**
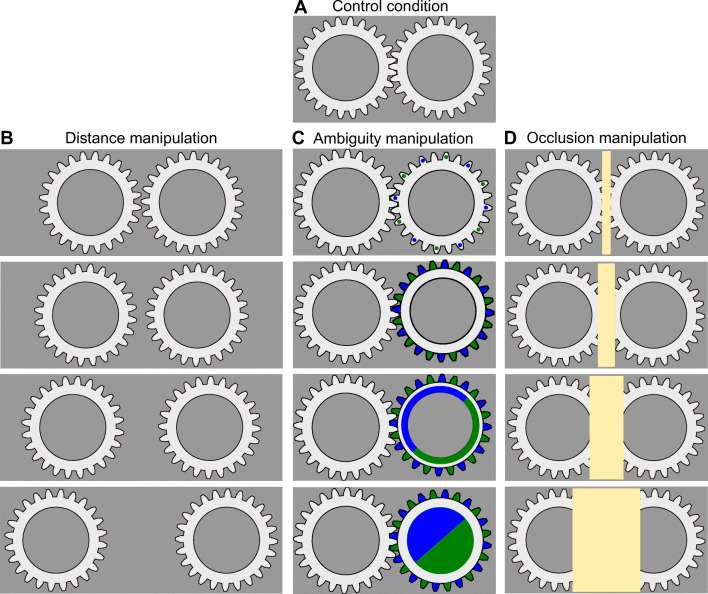
Experiment 1, schematic displays. (**a**) Control condition that allowed for a physical interaction between the gears and served as a common baseline for all stimulus manipulations. (**b**) Distance manipulation. (**c**) Ambiguity manipulation. (**d**) Occlusion manipulation

For the *control* condition, common to all comparisons, the gears were identical (plain faces, fully ambiguous) and interlocking (a distance of −8.13%/−0.569 dva); there was no occlusion (see Fig. 1a and Video 1). This condition represented a physically plausible interaction between the gears. Accordingly, it was a reference point and was included in the analysis of all experimental manipulations.

There were three experimental conditions, which manipulated *distance*, *ambiguity*, and *occlusion*(see Fig. 1b, c, and d). Each experimental manipulation had four levels plus the common *control* condition. For the *distance* condition, we manipulated the distance between the gears, which was −8.13%/−0.6 dva (*control* condition, the only condition that allowed for a physical interaction), 0%/0 dva (gears were touching but not interlocking), 8.13%/0.6 dva, 32.52%/2.3 dva, or 65.04%/4.6 dva (see Video 2). For the *occlusion* condition, the gears could always interact, but a rectangle covered the central part of the screen. It had a width of 0%/0 dva (absent, *control* condition), 8.13%/0.6 dva, 16.26%/1.1 dva, 32.52%/2.3 dva, or 65.04%/4.6 dva (see Video 3). For the *ambiguity* condition, the gears could always interact, but we disambiguated one of the gears by adding markings to its face (see Video 4). We used four levels of disambiguation plus a *control* condition with a plain gear face that was fully ambiguous. Please note that due to a programming error, the gears always rotated in the clockwise direction. This made no difference for fully ambiguous gears but caused a bias always towards clockwise rotation for the disambiguated gear.

Each experimental session consisted of 52 blocks. Each block lasted for one minute. The block order was randomized, and they were presented in ABBA order. Each experimental setting was presented four times.

The participants continuously reported on the perceptually dominant direction of rotation of the gears by pressing one of four corresponding arrow keys. *Left*, when both gears rotated counterclockwise. *Right*, when both gears rotated clockwise. *Up*, when the left gear rotated counterclockwise and the left clockwise (the overlapping part is moving up). *Down*, when the left gear rotated clockwise and the right gear counterclockwise (the overlapping part is moving down).

### Experiment 2. Walker on a ball

**Fig. 2:**
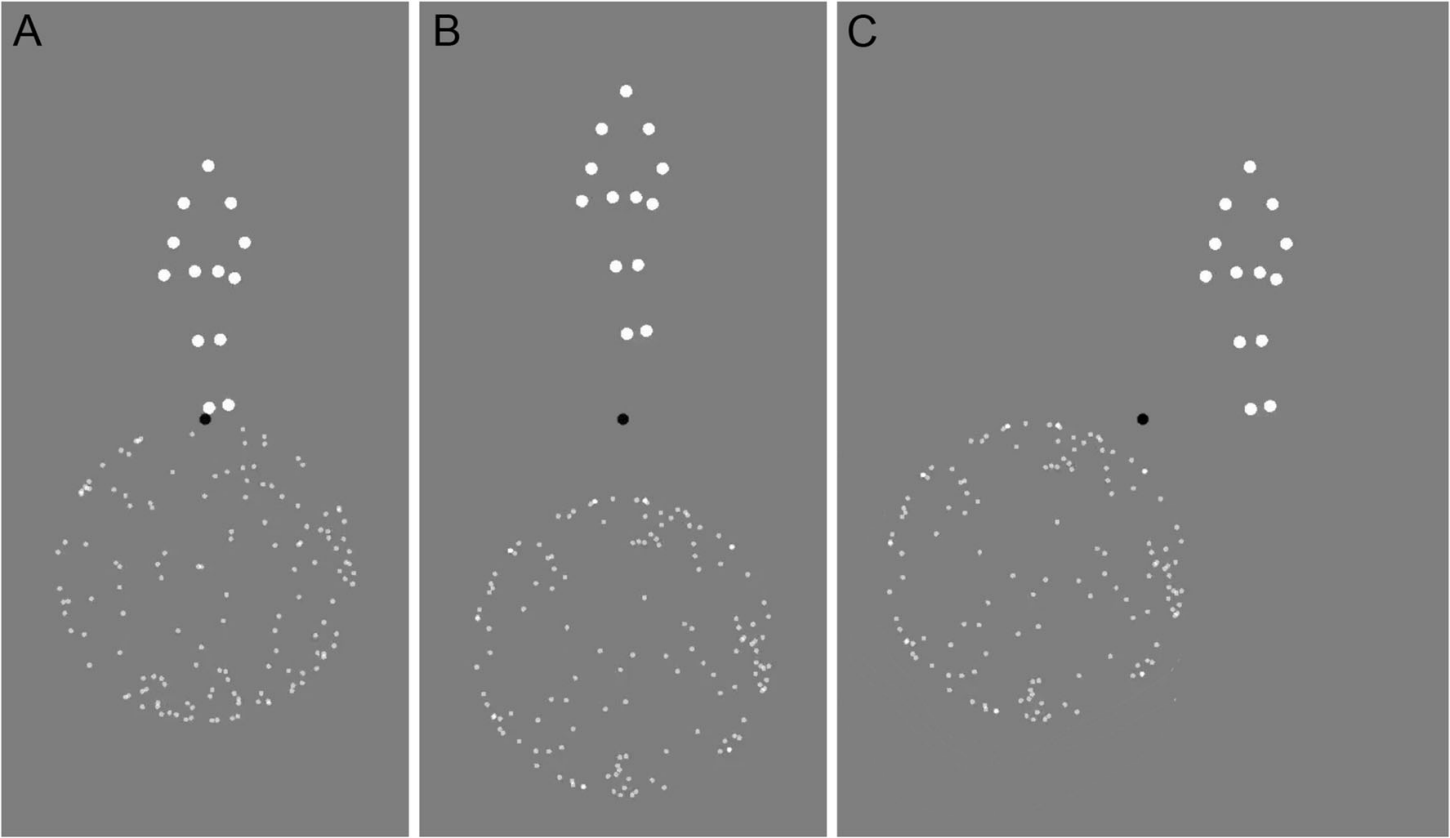
Experiment 2, schematic display. A walker on a ball. (**a**) Control condition. (**b**) Vertical displacement. (**c**) Horizontal displacement

The participants viewed a walker on a ball (a sphere), designed based on Jackson and Blake (2010; see Fig. 2). As in Experiment 1, each block lasted for one minute. The ball was represented by 140 dots (dot size was 0.1 dva) and rotated at a speed of 0.29 Hz/104°/s*—*a so-called “point-light rotating sphere.” The walker was represented by 13 dots (dot size was 0.25 dva) and was moving at the speed of 2 Hz*—*a so-called “point-light walker*.*” The walking sequence was based on Vanrie and Verfaillie (2004). Participants indicated whether their motion was physically congruent (i.e., their relative motion was consistent with a ball rolling under the walker’s feet). (Please see Video 5, which shows the walker and the sphere moving as if the sphere was rolling under the walker’s feet, and Video 6, which shows the walker and the sphere moving incongruently.)

For the *control* condition, common to all comparisons, the walker was located directly on top of the sphere (a distance of 0 dva; see Video 7). This condition resembled a physically plausible interaction between the walker and the ball and was designed after the walker and the sphere experiment in Jackson and Blake (2010).

There were five experimental conditions: a vertical distance, a horizontal distance, the walker ambiguity, the sphere ambiguity, and a single object report condition.

In the *vertical distance* condition, the walker and the ball were displaced vertically relative to each other (see Fig. 2b and Video 8). The distance was 0 dva (*control* condition), 1 dva, 2 dva, 3 dva, and 4 dva. For the *horizontal distance* condition*,* the walker and the ball were displaced horizontally relative to each other (see Fig. 2c and Video 9). The direction of the relative shift (i.e., whether the walker was shifted to the left and the ball to the right or vice versa) was systematically varied. The distance was 0 dva (*control* condition), 1 dva, 2 dva, 3 dva, and 4 dva. For both of these conditions, a physical interaction was possible only for the *control* condition.

In the *walker ambiguity* and the *sphere ambiguity* conditions, we added stereoscopic depth disambiguation cues to the respective object while keeping the other one fully ambiguous. Two projections—one for each eye—were rotated in the opposite direction around the vertical axis by 0° (fully ambiguous, *control* condition), ±0.25°, ±1°. For these two conditions, a physical interaction between the walker and the ball was always possible.

For the four experimental conditions above, participants continuously reported on both objects’ currently perceived relative motion (i.e., whether the direction of the walker movement and the sphere rotation were congruent).

In the *single object report* condition, they reported on one object only, either on the walker’s motion or on the ball’s rotation. Here, we systematically varied the horizontal displacement similar to the *horizontal distance* condition, with the distance 0 dva (*control* condition, the only condition that allowed for a physical interaction), 2 dva, and 4 dva. In addition, the direction of rotation of the ball was always disambiguated (stereo angle of ±1°). Participants were instructed before each block about which object they had to report on. They used the up or down arrow key for the walker moving towards or away from the participant and the left or right arrow keys to report on the rotation of the sphere. We deduced whether the walker’s motion was physically congruent, assuming that the sphere always rotated in the direction of the biasing cues, which was the case (see Fig. 6b). As in other conditions, participants reported continuously on their dominant visual perception.

*Vertical distance, walker ambiguity, and sphere ambiguity* conditions were repeated four times in an ABBA design. The *horizontal distance* and the *single object report* conditions, which were not part of the original preregistration, were repeated two times in an ABBA design.

### Statistical analysis

Statistical analysis was performed in R (Version 4.0.3; R Core Team, 2021) using the *tidyverse* family of packages (Wickham et al., 2019). Bayesian statistical analysis was performed using the *brms* package (Bürkner, 2018).

The data were fitted using four hierarchical models described below: (1) an independent perception model that assumed that participants’ perception was independent of stimulus manipulation and physical plausibility of an interaction between the objects (a.k.a. *independent perception* model); (2) model that assumed that changes in perception are proportional to changes in stimulus configuration (a.k.a. *stimulus-based* model); (3) a model that assumed that perception was different between stimulus configurations that allowed or did not allow for a physical interaction between two objects (a.k.a. *physics-based* model); (4) a hybrid interaction model that assumed that perception was differently influenced by stimulus configurations with and without a possibility of physical interaction (a.k.a. *hybrid-interaction* model).

In the model descriptions below, subscript *i* refers to individual observations and subscript *participant[i]* to an individual participant that the observation belongs to. Accordingly, *p*_*i*_ refers to the proportion of time participants reported counter-rotation (Experiment 1) or congruent motion (Experiment 2), whereas *μ*_*i*_ is the predicted mean of the beta distribution for that observation and *φ* is the precision parameter (see Bürkner, 2018) for details on reparametrization). Intercept terms are *α* and *α*_*participant[i]*_ for population-level and an individual participant, respectively. *S*_*i*_ and *NI*_*i*_ are, correspondingly, stimulus manipulation level (specific to a particular experiment and condition) and a binary flag that indicates whether that stimulus level prevented physical interaction (0 = *physical interaction is possible*, 1 = *physical interaction between two objects is not possible*). β_S_, β_NI_, and β_S×NI_ are, respectively, slope coefficients for stimulus manipulation level (Models 2 and 4), no-physical-interaction flag (Model 3), and their interaction (Model 4). We used default priors as suggested by the *brms* package, see Bürkner (2018) for details.

### Independent perception model (1)

The independent perception model assumed that the proportion of time (*p*_*i*_) participants reported counter-rotation (Experiment 1) or congruent motion (Experiment 2) reflected only their intrinsic sensory bias and was not influenced by the stimulus manipulation or physical plausibility of an interaction.

1 $$ {p}_i\sim BetaProportion\left({\mu}_i,\phi \right), $$

2 $$ logit\left({\mu}_i\right)=\alpha +{\alpha}_{participant\left[i\right]}, $$

3 $$ \alpha \sim Student\left(3,0,10\right), $$

4 $$ {\alpha}_{participant\left[i\right]}\sim Normal\left(0,10\right), $$

5 $$ \phi \sim Gamma\left(0.01,0.01\right). $$

### Stimulus-based model (2)

For the stimulus-based model, we assumed that gradual changes in the stimulus would lead to similar gradual changes in participants’ perception.

6 $$ {p}_i\sim BetaProportion\left({\mu}_i,\phi \right), $$

7 $$ logit\left({\mu}_i\right)=\alpha +{\alpha}_{participant\left[i\right]}+{\beta}_S\cdotp {S}_i, $$

8 $$ \alpha \sim Student\left(3,0,10\right), $$

9 $$ {\alpha}_{participant\left[i\right]}\sim Normal\left(0,10\right), $$

10 $$ {\beta}_S\sim Normal\left(0,1\right), $$

11 $$ \phi \sim Gamma\left(0.01,0.01\right). $$

### Physics-based perception model (3)

The physics-based model assumed that the participants would perceive stimuli differently if they either allowed or did not allow for physical interaction between two objects. For this purpose, we computed an additional variable *NI* (No physical Interaction) that was 0 for conditions that allowed for a physical interaction between the two objects, and for all other conditions, NI was 1.

12 $$ {p}_i\sim BetaProportion\left({\mu}_i,\phi \right), $$

13 $$ logit\left({\mu}_i\right)=\alpha +{\alpha}_{participant\left[i\right]}+{\beta}_{NI}\cdotp {NI}_i, $$

14 $$ \alpha \sim Student\left(3,0,10\right), $$

15 $$ {\alpha}_{participant\left[i\right]}\sim Normal\left(0,10\right), $$

16 $$ {\beta}_{NI}\sim Normal\left(0,1\right), $$

17 $$ \phi \sim Gamma\left(0.01,0.01\right). $$

### Hybrid interaction model (4)

For the hybrid interaction model, we added the *N*_*i*_ variable to the stimulus-based model so it would assume that the change in stimulus with or without the possibility for physical interaction will result in different perceptions for the participants.

18 $$ {p}_i\sim BetaProportion\left({\mu}_i,\phi \right), $$

19 $$ logit\left({\mu}_i\right)=\alpha +{\alpha}_{participant\left[i\right]}+{\beta}_s\cdotp {S}_i+{NI}_i, $$

20 $$ \alpha \sim Student\left(3,0,10\right), $$

21 $$ {\alpha}_{participant\left[i\right]}\sim Normal\left(0,10\right), $$

22 $$ {\beta}_s\sim Normal\left(0,1\right), $$

23 $$ {\beta}_{NI}\sim Normal\left(0,1\right), $$

24 $$ \phi \sim Gamma\left(0.01,0.01\right). $$

### Reported statistics

We characterized the population-level intercept and the fixed effect terms, denoted, respectively, as α and β in summary tables, using the samples from the posterior distribution. The intercept is expressed in the units of proportion (transformed via an inverse logit function). In contrast, fixed effects have units of log odds due to the logit link function that we converted to odds (via an exponential transformation) to facilitate their interpretation. For each term, we computed the mean and an 89% credible interval, a range that contains 89% of the probability mass based on values from the sampled posterior distribution (CI, also called compatibility interval). We chose to use an 89% CI because it is a prime number.

In addition, we refitted the stimulus-based model (see above) with stimulus manipulation as a *nominal* variable. We used posterior samples for pairwise comparison between the control condition (default stimulus configuration, leftmost on all plots) and each level of manipulation. For this, we computed change in a population-level posterior predicted proportion of reported counter-rotation (Experiment 1) or congruent motion (Experiment 2):

25 $$ \Delta  P=P\left({level}_i\right)-P(baseline), $$

26 $$ logit\left(P\left({level}_i\right)\right)=\alpha +{\beta}_{level}, $$

27 $$ logit\left(P(baseline)\right)=\alpha . $$

Values of Δ*P* below or above zero indicate that stimulus manipulation, respectively, decreased or increased the predicted proportion of counter-rotation/congruent motion. Posterior distribution of predicted difference Δ*P* expresses the same information as the posterior distribution for fixed effect slopes (β), which are used to compute it, but are easier to interpret as they express the change in the units of proportion rather than in log-odds (units of β due to logit link function). We characterized the change in predicted proportion Δ*P* using mean and 89% credible interval, which are reported on the upper x-axis of figures.

We compared all fitted models using a leave-one-out(LOO) information criterion (Vehtari et al., 2017). It computes an expected log predicted density (ELPD) that expresses expected out-of-sample deviance based on the posterior distribution of in-sample deviance (see Vehtari et al., 2017, for details). LOO information criterion is interpreted the same way as other information criteria, such as Akaike or Widely Applicable Information Criteria (AIC and WAIC, respectively), with lower values indicating better goodness-of-fit given the penalty for model complexity. We reported the difference in expected log predicted density (ΔELPD, mean ± standard error) relative to the best model (top model in each table, ΔELPD=0). In addition, we used ELPD to compute a relative weight for each model. The weights add up to 1, so a higher weight indicates a better relative estimated predictive ability of an individual model.

## Results

### Experiment 1: Gears

**Fig. 3:**
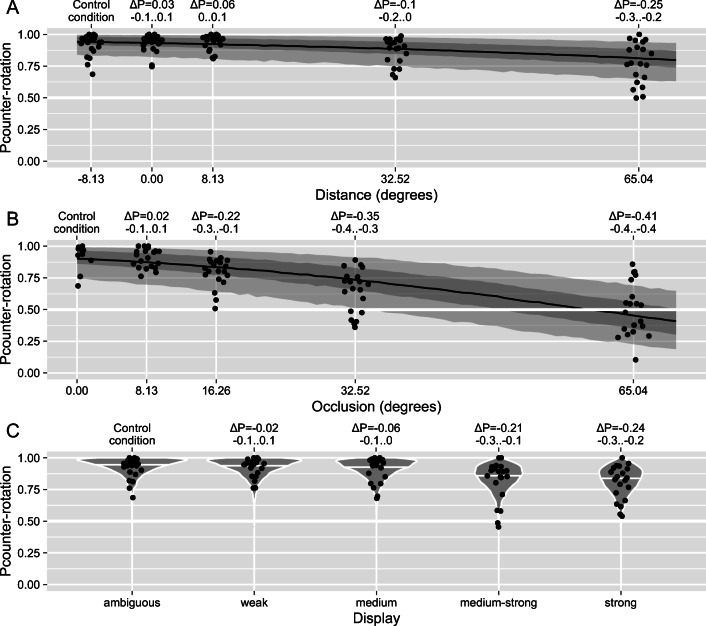
Experiment 1. The proportion of time participants reported counter-rotation as a function of (**a**) distance between the gears, (**b**) width of the occluding rectangle, and (**c**) ambiguity of the display. The leftmost *control condition* was the same for all three experimental manipulations. A, B) The black line, dark-gray, and light-gray stripes correspond to the median, 50%, and 89% credible intervals (CI) for the posterior predictions of the *stimulus-based* model. **c** The violin plots show the distribution of posterior predictions of the *stimulus-based* model; white horizontal lines depict the median of each distribution. **a–c** For all plots, dots depict individual observers. The upper *x*-axes show the median and the 89% CI for a change in the predicted proportion of corotation. See the Methods section for details

In our first experiment, we investigated the perception of two ambiguously rotating gears (see Fig. 1 and Video 1). Their default configuration—*control* condition when both gears were fully visible, had a plain face (no disambiguation cues), and were meshing (see Video 1)—allowed for physical interaction and served as a baseline for all the comparisons. Here, we found that the participants reported predominantly physically consistent counter-rotation(see Fig. 3 and intercept term α in Table 1).

**Table 1:** Experiment 1, statistical comparison of the four models for each stimulus manipulation

Model	ΔELPD	Weight	α [probability]	β [odds]
(A) Distance				
Stimulus-based	0	0.93	0.93 [0.91, 0.95]	0.9822 [0.98, 0.99]
Independent	−21.2±6.41	0.02	0.90 [0.86, 0.92]	–
Physics-Based	−21.3±6.08	0.00	0.91 [0.88, 0.94]	0.7686 [0.56, 1.04]
Hybrid-Interaction	−33.9±8.49	0.05	0.92 [0.90, 0.94]	1.0132 [0.96, 1.06]
(B) Occlusion				
Stimulus-based	0	1.00	0.91 [0.88, 0.93]	0.9632 [0.96, 0.97]
Hybrid-Interaction	−22.9±6.03	0.00	0.89 [0.87, 0.91]	0.9655 [0.96, 0.97]
Physics-Based	−54.1±7.07	0.00	0.76 [0.71, 0.80]	0.9979 [0.21, 4.96]
Independent	−54.3±7.09	0.00	0.76 [0.71, 0.80]	–
(C) Ambiguity				
Stimulus-based	0	0.97	0.93 [0.90, 0.96]	–
Independent	−17.6±5.17	0.00	0.89 [0.85, 0.91]	–
Physics-Based	−17.8±5.16	0.00	0.89 [0.85, 0.91]	1.0113 [0.21, 4.78]
Hybrid-Interaction	−35.4±8.21	0.03	0.90 [0.86, 0.92]	–

*Note. ΔELPD* the difference in expected log predicted density for a leave-one-out information criterion relative to the best model. *Weight* the relative weight of a model, based on ELPD values. Weights sum up to 1. *α (in the units of probability)* mean and 89% credible interval (CI) for the population-level intercept term of the model. *β (in the units of odds)* mean and 89% CI for the model term that reflects dependence either on changes in the stimulus (stimulus-based and hybrid-interaction models) or the possibility of physical interaction (physics-based model). The β value is not listed for ambiguity manipulation (C) because there was no single β coefficient for the stimulus-based model, as the stimulus was coded as a factor/categorical rather than a continuous variable. See the Methods section for details on reported statistics

As a comparison, we manipulated the *distance* between the gears, the extent to which the overlapping/interlocking part of the display was *occluded* from the observer, and the *ambiguity* of one of the gears. For all stimulus manipulations, we fitted the data using four hierarchical Bayesian models that (1) assumed that participants’ perception is independent of stimulus manipulation (*independent perception* model); (2) model that assumed that changes in perception are proportional to changes in stimulus configuration (*stimulus-based* model); (3) model that assumed that perception depended on whether a stimulus configuration allowed for a physical interaction between two objects (*physics-based* model); (4) and a model that assumed that stimulus configuration with and without a possibility of a physical interaction resulted in a different dependence on the stimulus changes (*hybrid-interaction* model). For details on models and reported statistics, please refer to the Method section.

In the *distance* experimental manipulation, we systematically varied the separation between the gears. Importantly, only the control condition (distance −8.13%/−0.6 dva) produced an overlap that allowed for the interlocking and, therefore, for an interaction between the gears. For all other distances, gears either touched but could not properly mesh (a distance of 0%/0 dva) or were separated by a gap that precluded their interaction (8.13%/0.6 dva, 32.52%/2.3 dva, or 65.04%/4.6 dva). Thus, a physics-based interaction model would predict a qualitative change in perception between the control condition and other distance levels. However, we observed a gradual effect of the distance on the participants’ perception (Fig. 3a) that was best explained by a *stimulus-based* model (see Table 1a).

Next, we examined how perception was affected by a rectangle that occluded the overlapping region of the two gears. We used this manipulation because prior work showed that occlusion facilitates the physics-consistent counter-rotation of two touching spheres (Gilroy & Blake, 2004). To this end, we placed a rectangle that covered the interlocking part of the gears and systematically varied its width (see Fig. 1 and Video 3). We found that the perception of counter-rotation was equally dominant for a small occluding rectangle as for the fully visible interlocking gears (*control* condition). However, the dominance of counter-rotation was significantly reduced for the larger rectangles (see Fig. 3b), even though the gears remained at the same locations, thus were technically interlocking and could interact. Again, as for the *distance* manipulation, it was the *stimulus-based* model that provided the best explanation for the data (Table 1b).

Finally, we studied the effect that the disambiguation of one of the gears had on the perception. Prior work showed that *perceptual coupling*(co-dependence of perceptual states) of the two bistable displays is reduced when one of the displays is disambiguated (Grossmann & Dobbins, 2003). These findings were interpreted as a purely perceptual effect, hence the name, and, if the dominance of counter-rotation in our experiment is mediated via the same or a similar mechanism, we would expect a similar decrease in counter-rotation for stronger disambiguation. In contrast, if the perception is determined by the rules of embedded physics of interaction, the disambiguation of one gear should have no effect, as the fully ambiguous gear can always accommodate the direction of rotation of the disambiguated one. To test this hypothesis, we disambiguated one of the gears by adding markings to its face using four levels of disambiguation (see Fig. S1 in the electronic supplementary material for the effectiveness of manipulation). The proportion of time our participants reported counter-rotation decreased gradually for stronger disambiguation levels (see Fig. 3c and Table 1c). In other words, disambiguation consistently biased perception of the single manipulated gear but decreased its perceptual coupling with the ambiguous one. Thus, as for other stimulus manipulations, our results were better consistent with the idea that participants’ perception is altered by stimulus changes rather than by affordance of a physics-based interaction.

To summarize the results of Experiment 1: For all experimental manipulations, we observed a gradual change in our participants’ perception rather than the abrupt qualitative change the physics-based heuristic would suggest.

### Experiment 2: Walker-on-a-ball

**Fig. 4:**
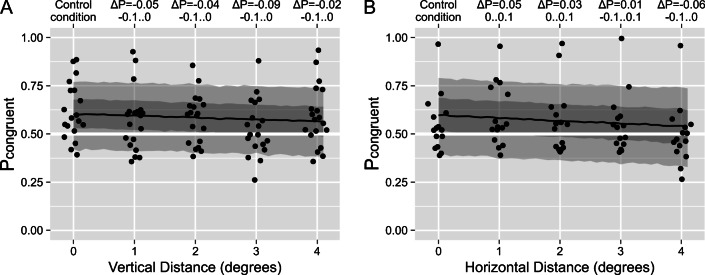
Experiment 2, distance conditions. The proportion of time the participants reported physically congruent perception as a function of (**a**) vertical and (**b**) horizontal distance between the walker-on-a-ball. The control condition on the left was the same for all experimental manipulations including those in Fig. 5. Each dot represents an individual observer. The black line, dark-gray, and light-gray stripes correspond to the median, 50%, and 89% credible intervals (CI) for the posterior stimulus-based model predictions. The upper *x*-axes show the median and the 89% CI for a change in the predicted proportion of congruent motion

In our second experiment, we used a walker-on-a-ball display, whose perception was reported to be biased towards a frictional—physics-consistent—interaction between the walker’s feet and the ball (Jackson & Blake, 2010). We designed our *control* condition based on the display in the original study with the walker being positioned directly on top of the sphere. Consistent with the prior work, we observed a moderate but robust bias toward the perception of the congruent relative motion (see *control* condition in Fig. 4). For our first four preregistered experimental manipulations—two that altered the distance between the walker and the ball and two that altered the ambiguity of the individual objects—the participants responded on *relative* motion (i.e., whether the motion was consistent with a person walking on a ball that rolls backward under their feet; see Video 5 and Video 6).

First, we sought to replicate the effect of the inter-object distance that decreased the counter-rotation for the gear displays in Experiment 1. To this end, we systematically varied both the vertical and horizontal distance between the walker and the sphere. Importantly, a physical interaction was possible only in the control condition when the walker was touching the sphere. Hence, the physical interaction model would predict a higher proportion of congruency for the control condition than for all other conditions. However, we found that the proportion of congruency reports remained virtually the same with only a minor decrease for large distances (see Fig. 4). Formally, the physics-based model had better predictive power for the vertical distance manipulation, whereas the stimulus-based one had a higher weight for the horizontal distance (see Table 2a and b). However, the expected log predicted density (ELPD, see Methods for details) was very similar across all models, including the perceptual independence model. For both manipulations and all models, the dependence (β in Table 2a and b) was only marginally different from 1.0.

**Table 2:** Experiment 2, statistical comparison of the three models for each stimulus manipulation

Model	ΔELPD	Weight	α [probability]	β [odds]
A) Vertical distance				
Physics-based	0	0.88	0.63 [0.56, 0.69]	0.8091 [0.68, 0.97]
Independent	−1.3±1.86	0.12	0.59 [0.53, 0.64]	–
Hybrid-Interaction	−1.4±1.42	0.00	0.61 [0.55, 0.66]	0.9999 [0.33, 3.12]
Stimulus-based	−1.8±1.43	0.00	0.60 [0.54, 0.66	0.9626 [0.91, 1.01]
B) Horizontal distance				
Stimulus-based	0	0.77	0.60 [0.51, 0.68]	0.9424 [0.89, 1.00]
Hybrid-Interaction	−0.3±0.36	0.00	0.60 [0.51, 0.68]	0.9734 [0.32, 3.05]
Independent	−0.6±1.56	0.01	0.57 [0.49, 0.65]	—
Physics-based	−0.8±1.69	0.22	0.57 [0.48, 0.65]	1.0325 [0.83, 1.28]
C) Sphere disambiguation				
Physics-based	0	1.00	0.64 [0.26, 0.90]	1.0195 [0.20, 5.19]
Independent	−0.2±0.2	0.00	0.65 [0.59, 0.70]	–
Hybrid-Interaction	−1.5±0.51	0.00	0.65 [0.59, 0.70]	0.9746 [0.32, 3.08]
Stimulus-based	−1.8±0.52	0.00	0.65 [0.59, 0.70]	0.9688 [0.78, 1.22]
D) Walker disambiguation				
Stimulus-based	0	1.00	0.62 [0.56, 0.68]	0.8254 [0.71, 0.96]
Hybrid-Interaction	−0.6±0.35	0.00	0.62 [0.56, 0.68]	0.9113 [0.30, 2.78]
Physics-based	−2.2±2.07	0.00	0.60 [0.24, 0.89]	1.0047 [0.19, 4.88]
Independent	−2.7±2.14	0.00	0.61 [0.55, 0.66]	–
E) Walker, single object report, the horizontal distance				
Independent	0	0.84	0.49 [0.40, 0.59]	–
Stimulus-based	−0.8±1.70	0.16	0.43 [0.30, 0.57]	1.1302 [0.91, 1.41]
Hybrid-Interaction	−0.8±1.57	0.00	0.43 [0.30, 0.58]	1.0592 [0.33, 3.37]
Physics-based	−1.3±2.13	0.00	0.43 [0.29, 0.58]	1.4920 [0.76, 2.98]

*Note. ΔELPD* the difference in expected log predicted density for a leave-one-out information criterion relative to the best model. *Weight* the relative predictive weight of a model (weight sum up to 1). *α (in units of probability)* mean and 89% credible interval (CI) for the population-level intercept term of the model. *β (in the units of odds)* mean and 89% CI for the model term that reflects dependence either on changes in the stimulus (stimulus-based and hybrid-interaction model) or in the possibility of physical interaction (physics-based mode)

**Fig. 5:**
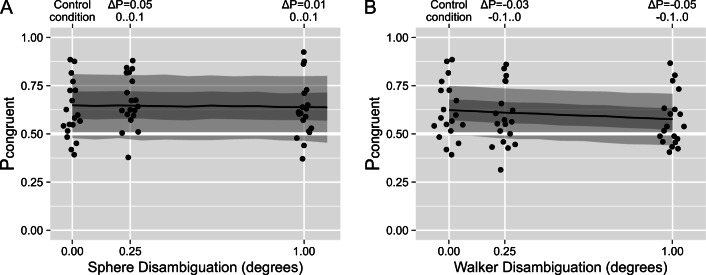
Experiment 2, ambiguity conditions. The proportion of time the participants reported physically congruent perception as a function of the strength of the disambiguation cues of (**a**) the sphere and (**b**) the walker. The control condition on the left was the same for all experimental manipulations, including those in Fig. 4. Each dot represents an individual observer. The black line, dark-gray, and light-gray stripes correspond to the median, 50%, and 89% credible intervals (CI) for the posterior stimulus-based model predictions. The upper *x*-axes show the median and the 89% CI for a change in the predicted proportion of congruent motion

Next, we systematically varied the ambiguity of the individual objects, as it was another experimental manipulation that gradually altered the perception of the ambiguous gears in Experiment 1. Again, we found a very weak effect of our manipulations (see Fig. 5) and a very similar goodness-of-fit for all three models (see Table 2c and d).

Taken together, the four experimental manipulations reported above show that visual perception of the walker-on-a-ball is unlikely to be based on or biased by physically plausible frictional interaction, as was presumed in the original study (Jackson & Blake, 2010). However, it also did not appear to reflect changes in stimuli, as the *perceptual-independence* model had a similar predictive power as more complex models. This indicates that some other perceptual or cognitive mechanisms were at play. One possibility was that our participants’ perception was biased by the fact that we explicitly asked them to judge relative motion congruency. This implied that they had to evaluate the relative motion of both objects together while contemplating which combinations would lead to a congruent perception. In turn, thinking about the congruent motion combinations could have biased perception via top-down attention (Gosselin & Schyns, 2003; Mossbridge et al., 2013). In this case, the bias towards congruent motion might be weakened, if the participants were asked to focus their attention and report about an *absolute* motion of a single object.

**Fig. 6:**
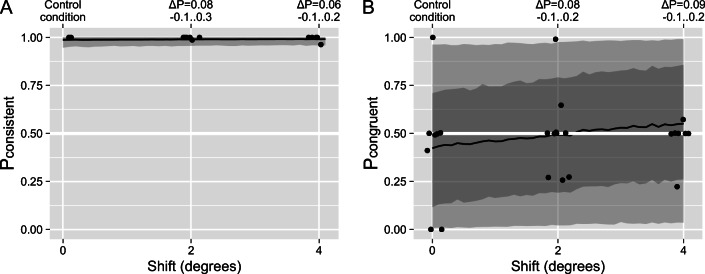
Experiment 2, single-object report condition. **a** The proportion of time the participants reported the sphere to rotate in the direction consistent with the disambiguation cues (i.e., the direction of rotation matched direction favored by stereo biasing cues, as a function of the horizontal distance between the objects). **b** The proportion of time the participants reported the direction of the walker’s motion that was physically congruent with the disambiguated direction of rotation of the ball (i.e., the ball rolled backwards under the walker’s feet, as a function of the horizontal distance between objects). The black line, dark-gray, and light-gray stripes correspond to the median, 50%, and 89% credible intervals (CI) for the posterior stimulus-based model predictions. The upper *x*-axes show the median and the 89% CI for a change in the predicted proportion of consistent (**a**) or physically congruent (**b**) rotation

To test this hypothesis, we used an experimental display with a strongly disambiguated rotation direction of the ball (stereo angle of ±1°) and a systematically varied horizontal distance between the walker and the sphere. The former ensured that the rotation of the ball was strongly biased, effectively, unambiguous, which we confirmed by asking the participants to report on the rotation of the sphere alone (see Fig. 6a, α = 0.9903 [0.98, 1.00], β = 1.0882 [0.86, 1.38]). When the participants were instructed to focus and report only on the walking direction of the walker, we found that the bias toward congruent motion was absent not only when the walker was shifted relative to the sphere but even when it was directly on top of it (see Fig. 6b). Moreover, the independent-perception model again had better predictive power than the more complex models (see Table 2e).

In short, we found that the participants were biased towards the perception of physically congruent motion when judging the relative motion of the two objects but the bias disappeared when they were to judge the motion of the walker alone. Apart from that, there was no further effect of any stimulus manipulation on their perception.

## Discussion

In this study, we investigated whether prior knowledge about a physically plausible interaction influences the perception of bistable objects. To this end, we examined the perception of two paired bistable displays—two ambiguously rotating gears and an ambiguous walker on an equally ambiguous rotating ball. In both cases, the displays were presented in a layout that would allow for physical interaction, but which was manipulated so that gradual changes in the visual stimulus would lead to either abrupt changes in the affordance of interaction or keep it constant. For both stimuli pairs and all employed manipulations, we found no evidence for perception to be biased by or based on a physically plausible interaction. Instead, we found that for bistable gears gradual changes in their appearance led to equally gradual changes in their perception and were best explained by a stimulus-based model. In the case of the walker-on-a-ball, all manipulations had little effect on participants’ perception, irrespective of a stimulus configuration or the opportunity for a physical interaction of two objects. Instead, their perception depended only on the task—that is, whether participants responded about the relative motion of two objects (in this case, the perception was biased towards physically congruent motion) or the absolute motion of the walker alone (here, its perception was independent of the rotation of the ball).

### Rotating gears

In the case of the ambiguously rotating gears, we used three manipulations. One—an increasing distance between the gears—was designed to produce a qualitative change with respect to the physics-based prior, as this manipulation made the interaction between the gears impossible. Conversely, the two other stimulus manipulations—disambiguation of one of the gears and occluding the overlapping/interlocking part of the gears—altered the display visually but always offered an opportunity for interaction. However, instead of abrupt changes in perception in the former case and unvarying one in the latter case, we observed very similar gradual changes in perception that mirrored similarly gradual changes of the stimulus. This continuous effect is incompatible with predictions of an embedded-physics-of-interaction theory. Instead, gradual changes of perception in response to stimulus changes are very similar to those observed in perceptually coupled multistable displays (Eby et al., 1989; Ramachandran & Anstis, 1983). In particular, the effects of both increasing distance and increasing disambiguation were consistent with prior work that used the same manipulations showing a decreasing correlation between perceptual states of multistable displays (Grossmann & Dobbins, 2003; Pastukhov, Zaus, et al., 2018b).

However, we must note that the actual perception for ambiguous gears was *opposite* to what would be expected from the perceptual coupling. The latter biases perception towards the *same* perceptual state in both objects; in our case this would be corotation. Yet, we observed strong and reliable *counter-rotation*. This discrepancy is likely to reflect the difference between the stimuli, although we can only speculate on what this difference could be. It is possible that opposite global perceptual states of the gears were driven by the similar motion of the individual teeth within the meshing region. Here, teeth from both gears could be perceptually coupled toward the same downward or upward motion. Thus, the same *local* motion would drive the opposite *global* rotation of objects. This would be consistent with diminished coupling in distance condition, as greater separation between gears would work against the local mechanisms, as well as in the occlusion condition that masks the local motion within the meshing region. In addition, one of the observers noted that vertical saccades biased the perception towards counter-rotation, again, suggesting that local, effectively, translational motion may dictate the global percept. Taken together, this would point to local sensory mechanisms of perceptual coupling and indicate that its strength may diminish for higher-order representations. Alternatively, it is possible that the robust counter-rotation of gears reflected participants’ internal model of interaction that biased the perception (Veto et al., 2018). However, it is not clear how such a model would produce quantitative rather than qualitative changes in perception in response to our stimulus manipulation.

### Walker-on-a-ball

The case of the walker-on-a-ball was particularly curious as all manipulations had little effect on participants’ perception, an outcome very different from that for ambiguous gears in Experiment 1 and other bistable displays (Grossmann & Dobbins, 2003). Moreover, we found that participants’ perception was biased not by stimulus manipulations but by the type of report they were required to make. When judging the relative motion of the two objects, their perception was biased towards physically congruent motion. However, this bias disappeared when they were reporting an absolute motion of the walker alone. We suspect that these changes in perceptual outcomes reflect participants’ preconceptions that work via top-down feedback. As noted above, the bias towards physically congruent motion was present only for relative motion judgments, for which we illustrated the concept using the examples of such congruent and incongruent *relative* motion. Therefore, it is most probable that when making a perceptual decision, the participants compared it to the template of congruency, involuntary biasing their perception (Gosselin & Schyns, 2003). Such an attentional modulation was reported to have a strong effect on the perception of single bistable displays (Hancock & Andrews, 2007; Hol et al., 2003; Kohler et al., 2008). Conversely, for a single object report, such a comparison to the template was unnecessary, leading to an unbiased perception. We believe that it is strong evidence for the cognitive rather than perceptual nature of the congruency-bias in the walker-on-a-ball display. It will be interesting to investigate whether a similar template-driven perception of relative motion also affects other multi-object displays, such as streaming-bouncing(Burns & Zanker, 2000).

In hindsight, it is also clear that “normal” perceptual coupling mechanisms were unlikely to explain the perception of the walker-on-a-ball displays. As noted above, perceptual coupling involves two nearby bistable objects that can be perceived in the *same* state. In this case, the interaction could work via lateral connections between spatially adjacent neural representations (Klink et al., 2009) or via a spatial “spill-over” of top-down feedback (Grossmann & Dobbins, 2003). However, all these individual neural representations are coding the same perceptual property, such as rotation, and could be expected to have such a direct relationship or to be influenced by the same top-down bias. In contrast, the biological motion of the walker and the rotation of the ball are very different types of motion and are represented in different regions of the visual cortex. Specifically, the region hMT+ is a prime candidate for the representation of the ball’s rotation (Brouwer & van Ee, 2007), whereas biological motion is associated with activity in the posterior portions of the superior temporal sulcus (Peuskens et al., 2005). Thus, the two representations are very different and are unlikely to be linked directly, as two identical representations of rotation would, and their interaction is likely to reflect a more indirect but more general mechanism of conscious perception.

### Lack of embedded physics of interaction

Returning to the main question of the study, we must conclude that our visual system does not readily rely on prior knowledge about the physical interaction of objects. The results of the current study and earlier work consistently fail to find evidence for it and, sometimes, even fail to reproduce earlier results (Pastukhov, Prasch, & Carbon, 2018a; Pastukhov, Zaus, et al., 2018b). Although we replicated earlier findings by Jackson and Blake (2010), we found that additional manipulations rule out a perception based on actual physical interaction.

In principle, the results of Experiment 1 could still allow for a crude prior of interaction that biases the perception of two gears irrespective of the *finer* details of the configuration. This would not be a “true” physics-based prior, but it is easy to see why using such “true” prior knowledge is problematic, as it requires either very precise information about the objects or many assumptions in place of this information. For example, a frictional interaction between the two spheres (Gilroy & Blake, 2004) would only work if the two spheres were perfectly aligned. Even a small, effectively imperceptible gap between them or a slight shift of one of them in depth would prevent them from touching each other and therefore render frictional interaction impossible. Thus, physical interaction is not given even if the objects do *appear* to be at compatible positions. It is likely advantageous to use local single object priors to resolve uncertainty rather than to impose a strong group-based prior that is more likely to be wrong than right. During our daily lives, we are unlikely to frequently encounter such interacting objects with high uncertainty about their motion, such as bistable ones. In most cases, their motion (perceptual states) would be strongly biased by sensory information, reducing the need for the overarching interaction prior.

We must stress that our results do not imply that knowledge about statistical regularities of the outside world is not embedded in perception. On the contrary, as described in the introduction, our perception draws heavily on such statistical knowledge that must, at least to some degree, reflect the laws of physics that describe these regularities. However, we must remember that our knowledge of these regularities at a cognitive level may exceed information embedded in sensory level processing. Even when prior knowledge can be described and leads to robust predictions, such as for “slow prior” in motion perception (Weiss et al., 2002), its actual implementation in the brain, while functionally equivalent, may rely on a combination of several regularities reflected in neural processing rather than on a single prior (Rideaux & Welchman, 2020).

In short, our study also acts as a warning that it is easy to overestimate the amount and the nature of statistical knowledge embedded in our perception.

## Conclusions

To conclude, we demonstrated that for two bistable objects—ambiguously rotating gears (Experiment 1) and walker-on-a-ball displays (Experiment 2)—perception was not predicted by physics-based prior knowledge of the interaction. Instead, we report that the perception of the two displays appears to be mediated by a different neural mechanism. Specifically, the mechanisms of perceptual coupling are likely to govern the perception of ambiguously rotating gears. However, a cognitive mechanism, most likely involving selective attention, biased the perception of the walker-on-a-ball.

## Supplementary Information

ESM 1 (DOCX 57 kb)

Video 1. Experiment 1, gears. Control condition that allowed for physical interaction. (MP4 608 kb)

Video 2. Experiment 1, gears. Distance condition. (MP4 627 kb)

Video 3. Experiment 1, gears. Occlusion condition. (MP4 468 kb)

Video 4. Experiment 1, gears. Ambiguity condition. (MP4 804 kb)

Video 5. Experiment 2, walker on a ball. An example of congruent perception. Please use anaglyph glasses. (MP4 385 kb)

Video 6. Experiment 2, walker on a ball. An example of incongruent perception. Please use anaglyph glasses. (MP4 391 kb)

Video 7. Experiment 2, walker on a ball. Control condition that allowed for physical interaction. (MP4 403 kb)

Video 8. Experiment 2, walker on a ball. Vertical distance condition. (MP4 396 kb)

Video 9. Experiment 2, walker on a ball. Horizontal distance condition. (MP4 568 kb)
