# Supplementary material for: When perception is stronger than physics: Perceptual similarities rather than laws of physics govern the perception of interacting objects
Source: Atten Percept Psychophys. 2021 Oct 18;84(1):124–37. doi: 10.3758/s13414-021-02383-1 (PMC8522868; doi:10.3758/s13414-021-02383-1)
Supplement: Supplementary file 1 — (DOCX 57 kb) [file 13414_2021_2383_MOESM1_ESM.docx]

# Experiment 1


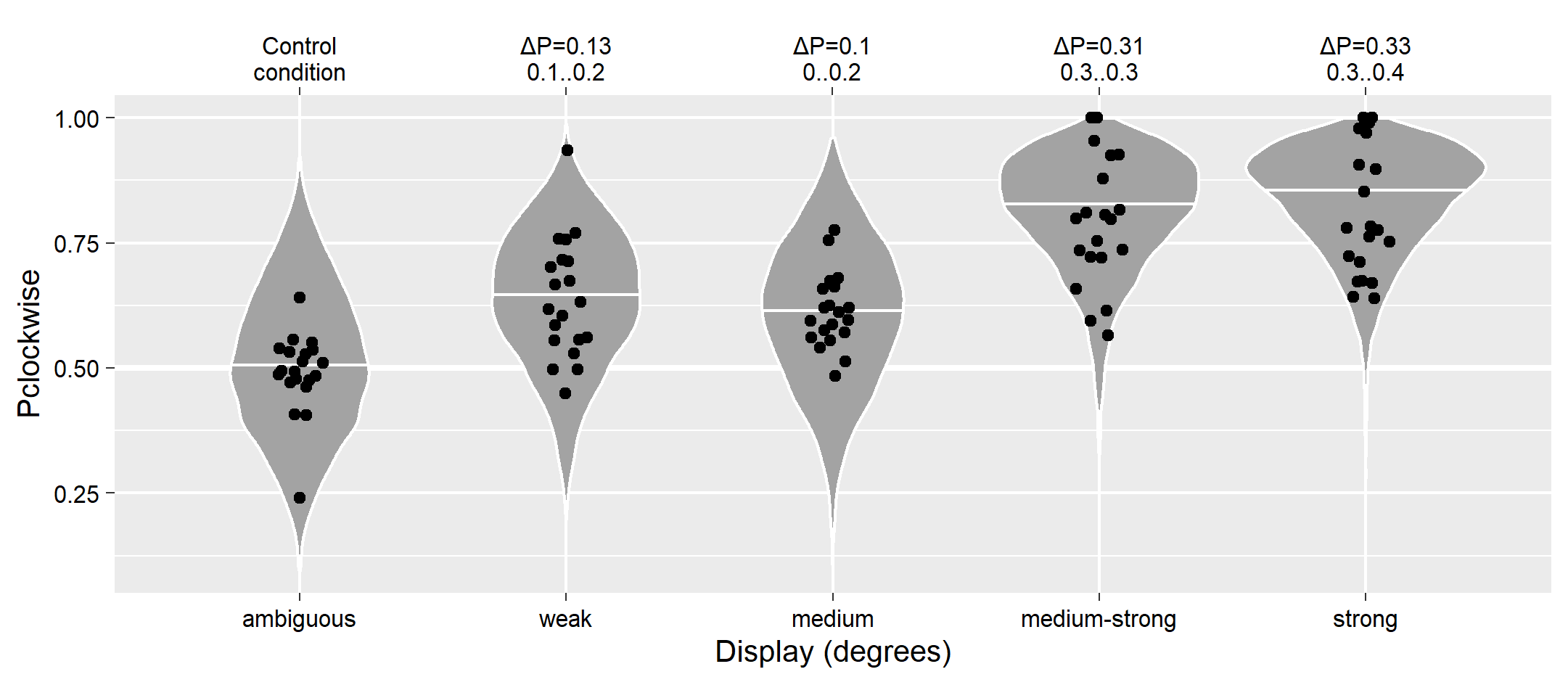


**Fig. S1** Experiment 1, the proportion of time the participants reported clockwise rotation for a disambiguated gear as a function of its ambiguity. Upper *x*-axes show the median and 89% credible interval for a change in the predicted proportion of clockwise rotation. See the Methods section for details
